# Seroprevalence and transmission of Hepatitis B virus among delivering women and their new born in selected health facilities, Addis Ababa, Ethiopia: a cross sectional study

**DOI:** 10.1186/1756-0500-7-239

**Published:** 2014-04-15

**Authors:** Dessie Tegegne, Kassu Desta, Belete Tegbaru, Tesfaye Tilahun

**Affiliations:** 1 Department of Biomedical Science, College of Health Sciences, Samara University, Samara, Ethiopia; 2 Department of Medical Laboratory Sciences, School of Allied Sciences, College of Health Sciences, Addis Ababa University, Addis Ababa, Ethiopia; 3 Ethiopian Health and Nutrition Research Institute (EHNRI), Addis Ababa, Ethiopia

**Keywords:** Hepatitis B virus, Seroprevalence, Risk factor, Delivering women, Cord blood

## Abstract

**Background:**

Hepatitis B Virus is a major public health problem worldwide. In 2012 alone, over 350 million chronic carriers and 1. 2 million annual deaths were occurred. Hepatitis B Virus causes 60 to 80% of the world’s primary liver cancer and nearly 90% infants infected due to vertical transmission are at higher risk of developing chronic liver disease and cancer. Hence determining the burden of maternal and neonatal Hepatitis B Virus infection is a priority.

**Methods:**

A cross sectional study was conducted from July – September 2012 at St. Paul’s Hospital Millennium Medical College and Selam Health Center, Addis Ababa, Ethiopia. Blood samples from delivering mothers (n = 265) and their corresponding cords (n = 265) were collected. A pretested questionnaire was used to collect data. Hepatitis B Virus surface antigen was detected using Enzyme Linked Immunosorbent Assay. Frequency analysis and logistic regression test was used to identify the potential risk factors associated with Hepatitis B Virus positivity using SPSS Version -15.

**Results:**

A total of 265 delivering women with the mean age of 25.8 years were enrolled in the study. Of these delivering women, 8 (3.0%) of mothers were positive for Hepatitis B Virus surface antigen, whereas 6 (2.3%) of cord bloods were positives with 75% concordance rate of exposed infants with sero-positive mothers. However, only one maternal positive case was observed for Hepatitis B e Ag test. Only 11% of the mothers know their Hepatitis B Virus status. Of the total mothers assessed for possible risk factors, 69 (26%) had only one type, while 161 (60.8%) had multiple exposure factors such as ear pricing, history of tribal marks, abortion, multiple-sexual partner and history of surgical procedures experienced from high to low frequency. The remaining 35 (13.2%) of the participants had not experienced possible risk factors.

**Conclusion:**

Though the maternal positivity rate was low, the rate of positivity in cord bloods was almost equal to those infected mothers. Therefore, screening of pregnant mothers and vaccination of infants could help to reduce the transmission. To minimize the higher overall risk exposure status of mothers, increasing awareness and intensive public health education is also recommended.

## Background

Hepatitis B virus (HBV) infection is one of the leading causes of liver diseases causing serious public health problem worldwide. It is 50 – 100 and 10 times more infectious than human immune deficiency virus (HIV) and hepatitis C virus (HCV), respectively. Many of the carriers are not realizing that they are infected with the virus and thus HBV is referred as a “silent killer” [1].

The virus is found in all body fluids and therefore, transmission could be either vertically (from infected mother to the infant or to the child) or horizontally (sexually and/or contact with different body fluids) [2,3]. However, mother to child transmission (MTCT) of HBV is of great importance for two main reasons as it is numerically important mode of transmission and the earlier an individual is infected the more likely to develop chronic disease [4]. In highly endemic areas, up to 75% of chronic carriers acquire the infection through vertical transmission [5]. The transmission and infection during infancy increases the chance to develop chronic disease to 90%, while adults usually acquire acute hepatitis B and recover, and hence the chance is 6 to 10% only [6].

In most developing countries with high HBV prevalence, intrauterine/transplacental transmission can lead to coagulation defects, postpartum hemorrhage, organ failure, high maternal mortality and poor outcomes of their newborns such as still births, neonatal deaths, acute and chronic liver disease, hepatocellular carcinoma and increased premature delivery [7].

According to recent WHO report, one- third of the world’s populations have serologic evidence of past or present HBV infection. Of these, about 350 million are chronic carriers [8,9], and 1. 2 million die annually from chronic hepatitis, cirrhosis and hepatocellular carcinoma [10]. HBV causes 60 to 80% of the world’s primary liver cancers [11]. The reported prevalence of chronic HBV infection varies widely, from high (≥8%, in Africa, Asia, and the Western Pacific countries) to intermediate (2-7% in Southern and Eastern Europe), and low (<2%, in Western Europe, North America and Australia) [12].

Studies conducted on the mechanism of intrauterine infection of HBV in China showed 81.4% positivity rate of hepatitis B surface antigen (HBsAg) in placentas from 59 HBsAg- positive mothers [13]. However, a study from Nigeria showed 51.6% vertical transmission rate from 8.3% HBsAg positive mothers [14]. In Ethiopia, three studies [Jimma (South West), and DebreTabor and BahirDar (North West)] showed a seroprevalence of 3.7%, 5.3% and 3.8%, among pregnant women, respectively [15-17].

In spite of this, routine antenatal screening for HBV infection and vaccination is lacking in many Ethiopian health facilities [16,18]. Moreover, no recent published data is seen on the maternal and neonatal HBV infection in Ethiopia generally and in Addis Ababa particularly. Therefore, this study was aimed at finding out the burden of HBV and asses possible risk factors among delivering women and their new born in some selected health facilities in Addis Ababa, Ethiopia.

## Methods

### Study design, setting & study period

A cross sectional study was conducted from July-September 2012 among consecutively recruited 265 delivering women and their respective infant cord samples from two selected health facilities in Addis Ababa, Ethiopia [St. Paul’s Hospital Millennium Medical College and Selam Health Center]. Both health facilities give anti-natal care, delivery, post-natal care, and any fetomaternal related services including anti retro viral therapy (ART) within the facility and from referral cases. The monthly delivery rate of the two facilities at the time of the study was, 150 and 40, respectively.

### Population

All pregnant women who came for delivery during the study period were used as source population. Those women who were delivered during the study period at the two study health facilities and fulfill the inclusion criteria were considered as a study population.

### Sample size calculations and data collection

The sample size was calculated based on single sample size estimation [19]. The prevalence rate (p) was taken as 6.1% from a previous study conducted [20] with the precision of 3.5%. From this, a sample size was calculated as 265 (including 10% non response rate) with 265 corresponding cord samples. Midwifes and nurses were trained to collect data (using a pre-tested well structured questionnaire). Five milliliters of whole blood samples from mothers and the corresponding cords were collected aseptically using ethylene diamine tetra-acetate (EDTA) test tubes (BD, USA). Plasma samples were separated and stored in a refrigerator (-20 degrees) until tested for HBsAg using Enzyme Immuno Sorbent Assay (ELISA) [DIALAB, Germany] and for Hepatitis B e Ag (HBeAg) [Insight HBeAg rapid test kit, [TULIP DIAGNOSTICS, Germany].

### Data analysis

The data were entered in to SPSS (version 15) and double checked before analysis. Descriptive statistics (means, percentages or frequency) were calculated and the associations were assessed by using the logistic regression. Significance associations were tested using a p-value < 0.05.

### Ethical clearances

Ethical clearances were obtained from the “Department of Research and Ethical Review Committee” of the Medical Laboratory Sciences, School of Allied Health Sciences, College of Health Sciences, Addis Ababa University and St Paul’s Hospital Millennium Medical College institutional review board. Additional permission was also obtained from the health facilities. The purpose of the study was explained and consent was obtained from each participant, and those with informed consent were enrolled in the study. Test results were given to the clinicians for further clinical management and follow up.

## Result

### Socio demographic characteristics of study participants

A total of 265 delivering women and their respective infant cord blood samples were collected [221 (83%) from St. Paul’s Hospital Millennium Medical College and 44 (17%) from Selam Health Center] in the period of July 2012 to September 2012. The mean age was 25.8 years (Range: 15–40 years). Most of them (77%) were living in Addis Ababa and 91.3% of them were married. Of all, 38% and 8.5% of them had a primary and tertiary education, respectively (Table 1). Only 11% of the mothers know about their HBV status in their anti-natal care (ANC) follow-up.

**Table 1:** Socio- demographic characteristics of delivering women at St Paul’s Hospital Millennium Medical College and Selam health center Addis Ababa, Ethiopia (July-September, 2012)

**Demographic characteristics**	**Frequency**	**Percent (%)**
**Age (in years)**		
15-19	20	7.6
20-24	84	31.7
25-29	105	39.6
30-34	36	15.6
35-40	20	7.6
**Marital status**		
Married	242	91.3
Unmarried	22	8.3
Divorced	1	0.4
Widowed	0	0.00
**Education level**		
Illiterate	83	32
Primary	101	38
Secondary	58	21.5
Tertiary	23	8.5
**Monthly income (In Ethiopian Birr)**		
<500	91	34.3
500-1000	116	43.8
1001-1500	22	8.3
1501-2000	11	4.2
>2000	25	9.4
**Ethnicity**		
Oromo	90	34.0
Gurage	80	30.2
Amhara	69	26.0
Tigre	10	3.8
Siltie	14	5.25
Others	2	0.75

### Maternal and neonatal cord blood Hepatitis B virus positivity

Of the total 265 study participants tested, 8 (3.0%) [95% CI: 2.7 - 3.4] of mothers and 6 (2.3%) [(95% CI: 2.0 - 3.5] of infant’s cord blood samples were positive for HBsAg [2 infants per 100 mothers]. Of the infant cord blood samples, 6 (75%) of them had similar results to their infected mothers, while 2 (25%) of them had negative results. From all samples tested for HBeAg, only one case (12.5%) was positive. At least one HBsAg positive result was observed in each age group and in every education category. However, only house wives and governmental employees had more HBsAg positive results. Except the facility, where the participants are recruited, other socio demographic characteristics were not significantly associated with HBV positivity (p-value > 0.05).

Although no statistically significant difference among the age groups, the highest and lowest prevalence was observed in the age groups of 35–40 years (10%), and 25–29 years (1.9%), respectively (Table 2).

**Table 2:** Maternal HBV status with demographic charactersticies at St Paul’s Hospital Millennium Medical College and Selam Health Center Addis Ababa, Ethiopia (July-September, 2012)

**Demographic characteristics**		**HBV status of mothers**		**Cr. OR**	**Adj. OR**
		**Positive**	**Negative**		
		**N (%)**	**N (%)**		
**Address**	Addis Ababa	5 (1.9%)	199 (75.1%)	0.85 (0.35-11)	0.2 (0.5-1.2)
	Out of Addis Ababa	3 (1.12%)	58 (21.88%)	1	1
**Facility**	St PMMCH	8 (3.02%)	213 (80.37)	1.2 (1.1-13)*	1.7 (1.3-22)
	SHE	0 (0%)	44 (16.6%)	1	1
**Age group**	15-19	1 (0.38%)	19 (7.2%)	2 (0.2-10)	8.3 (0.3-23)
	20-24	2 (0.76%)	82 (30.9%)	4.6 (0.6-15)	12 (0.9-35)
	25-29	2 (0.76%)	103 (38.9%)	5.7 (0.8-43)	9 (0.8-102)
	30-34	1 (0.38%)	35 (13.2%)	4 (0.33-46)	7 (0.4-122)
	35-40	2 (0.76%)	18 (6.8%)	1	1
**Education**	Illiterate	4 (1.51%)	81 (30.55%)	3.5 (0.3-24)	6 (0.17-235)
	Primary	3 (1.13%)	98 (37.0%)	1.7 (0.08-14)	4 (0.1-147)
	Secondary	1 (0.38%)	56 (21.13%)	1.2 (0.2-11)	1.7 (0.03-73)
	Tertiary	0 (0.0%)	22 (8.30%)	1	1
**Monthly income (in Birr)**	<500	3 (1.13%)	88 (33.23%)	0.15 (0.6-8)	0.3 (0.04-3)
	500-1000	2 (0.76%)	114 (43%) 20	0.2 (0.09-3)	0.4 (0.01-3.4)
	1001-1500	2 (0.76%)	(7.54%)	1.3 (0.56-7)	1.6 (0.14-18)
	15.1-2000	0 (0.00%)	11 (4.15%)	0.13 (0.8-9)	0.2 (0.01-28)
	> 2000	1 (0.38%)	24 (9.05%)	1	1
**Marital status**	Married	7 (2.64%)	235 (88.7%)	1.65 (0.75-3)	3 (1–28)
	Single	1 (0.38%)	22 (8.3%)		1

*Adj. OR*, Adjusted odds ratio; *Cr. OR*, Crude odds ratio; *p-value < 0.05; *St. PMMCH*, St. Paul’s Hospital Millennium Medical College; *SHC*, Selam Health Center; *1*, Reference value; *N*, Number.

Despite small number of positive cases, HBV infection showed a decrease trend with education [(1.5%) from illiterates, 1.13% from primary, 0.38% from secondary and (0.0%) from tertiary educated participants].

### Risk factors and HBV positivity

Among the possible risk factors for HBV infection assessed, the highest possible risk factor was ear pricing (69%) and the lowest was history of surgical procedures (9%). The specific distribution of risk factors and their association with the HBV positivity is given on Table 3. Twenty (7.5%) of the total delivering women were HIV positives, of whom 16 (80%) of them were on ART. However, none of them were positive for HBV. Generally, in this study, 35 (13.2%) of the participants had no any kind of possible risk factor for exposure. Only 26% had one type of exposure and about 60.8% of the study participants had multiple exposure history. However, among those with multiple exposures, 4 of them were HBV positives. None of the expected possible risk factors were significantly associated with the status of HBV positivity (p-value >0.05), which might be due to small number of HBV cases.

**Table 3:** Association of possible potential risk factors and maternal HBV status at St Paul’s Hospital Millennium Medical College and Selam health center Addis Ababa, Ethiopia (July 2012 - September 2012)

**Risk factors**	**HBsAg positive**	**HBsAg –Ve.**	**Cr. OR.**	**Adj. OR.**
	**N (%)**	**N (%)**		
**Dental procedure**	Yes: 2 (0.76%)	30 (11.32%)	0.4 (0.07 - 2.0)	2 (0.25-17)
	No: 6 (2.26%)	227 (85.66%)	1.00	1
**Surgical procedure**	Yes: 3 (1.14%)	21 (7.90%)	1.2 (0.03 - 1.7)	3.2 (0.56-18)
	No: 5 (1.88%)	236 (89.0%)	1.00	1
**Tattooing**	Yes: 4 (1.50%)	53 (20.0%)	0.26 (0.6 - 1.07)	1.2 (0.3-2.4)
	No: 4 (1.50%)	204 (77.0%)	1.00	1
**Abortion**	Yes: 2 (0.76%)	42 (15.84%)	0.6 (0.11 - 3.0)	1.6 (0.2-13)
	No: 6 (2.26%)	215 (81.14%)	1.00	1
**Ear piercing**	Yes: 6 (2.26%)	177 (66.8%)	0.74 (0.15 - 3.7)	0.6 (0.07-4)
	No: 2 (0.76%)	80 (30.20%)	1.00	1
**Blood received**	Yes: 2 (0.76%)	10 (3.77%)	1.1 (0.01- 1.2)	7 (0.8 - 69)
	No: 6 (2.26%)	247 (93.2%)	1.00	1
**Multigravida**	Yes: 4 (1.51%)	102 (38.5%)	0.67 (0.16 - 2.7)	1.05 (0.2-5.6)
	No: 4 (1.51%)	155 (58.5%)	1.00	1
**Multiple sexual partner**	Yes: 2 (0.76%)	37 (14.0%)	0.51 (0.1 - 2.6)	1.5 (0.2-10)
	No: 6 (2.26%)	220 (83.0%)	1.00	1
**Caesarian section**	Yes: 4 (1.51%)	62 (23.42%)	0.32 (0.08 - 1.3)	1.8 (0.36-9)
	No: 4 (1.51%)	195 (73.56%)	1.00	1

*P-val.*, P-value; *Adj. OR*, Adjusted odds ratio; *Cr. OR*, Crude odds ratio; -*ve.*, Negative; *HBsAg*, Hepatitis B surface antigen; *1*, Reference value; *N*, Number.

## Discussion

HBV is the common cause of liver cancer worldwide with 350 million chronic carriers and 1.2 million annual deaths [8,10]. It is 50 – 100 and 10 times more infectious than HIV and HCV, respectively [1]. Vertical transmission from infected mothers to their infants occurs through intrauterine transmission. This way is the major source of infection in many endemic areas [21]. About 90% of the infected infants develop chronic carrier state (of which 25% develop cancer) compared to adults with below 10%.

Centers for Disease Control and Prevention (CDC) recommended screening of all pregnant women for HBV and provision of prophylaxis for those who are positives [22]. If mothers are not screened at ANC and/or with no documented results, the screening should be done on delivering mothers as soon as possible; and vaccine to be given for infants within 12 hours of delivery [23]. Despite this, in this study, only 11% of the mothers know their HBV status, which indicates the screening should be strengthened in these health facilities during their ANC follow-up.

In our study, the overall prevalence among delivering women was 3.0% for HBsAg and only one case for HBeAg. This makes the study sites as an intermediate endemic area (2–7%) for HBV infection according to WHO criteria [24,25]. Our finding is in agreement with the previous reports of HBsAg infection rate among pregnant women in Western (3.7%) and North Western (3.8%) Ethiopia [15,17]. In contrast, lower prevalence rates were observed from Saudi Arabia (1.6%) and Libya (1.5%) [26,27]. Moreover, higher infection rates were also observed in Sudan (5.6%), Yemen (10%) and Niger (5.6%) [28-30]. Higher prevalence rates as high as 10% were reported from India (10%), Nigeria (11%), Yemen (10%) and Mali (8%) [14,30-32], respectively. The variations observed compared to this study could be due to the methods used, geographical variations, sample size and the study design. Moreover, unlike ours, some reports such as from those Asian countries (India and China) were from the highest prevalence regions of WHO criteria [31]. However, those earlier studies from urban Ethiopia also showed higher prevalence (in 1988), which might be due to lower awareness of the population towards HBV infection in that period [16].

The assessed HBV infection in neonates, due to vertical transmission (cord blood) with a prevalence of 2.3% and 75% of concordant result with mother’s positivity might directly showed the increased exposure of infants from their mothers through intrauterine transmission. This might lead to a higher chance (up to 90% chronic carrier state) of increasing in the development of liver cancer among these chronically infected children in their lifetime. In contrast to our findings of higher concordance with the infants, slightly lower rates of concordance were reported from India 50% [31], Nigeria 51.6% [14], and Libya (60.9%) [27]. These observed lower rates might be due to effective ANC screening, use of vaccine and provision of prophylaxis as the major prevention mechanisms, unlike ours with a very low ANC screening coverage (11%).

In most epidemiological studies on HBV infection, there has been a link between age and the acquisition of HBsAg that indicates, age of acquiring of the infection as one of the major determinant factor for HBsAg positivity [27]. In our study, although not statistically significant, higher prevalence (10%) and lower prevalence (1.9%) was observed in the age groups of 35–40 and 25–29 years, respectively. Similar findings were reported from Saudi Arabia that showed an increase in prevalence with age and higher prevalence with age groups greater than 25 years compared to those less than 25 years [26].

Although level of education is known as the determinant factor for HBV infection with a reverse pattern, which might be due to the level of awareness, so many findings like our finding showed no significant differences among different study populations such as those from Nigeria [14,32], Sudan [28], Mali [33] and Zimbabwe [34].

Some factors are known to be directly or indirectly lead to exposure for HBV infection [14,15]. In our study, from all delivering women, the highest percentage 183(69%) of them had ear pricing history of whom 6 (2.3%)) were from HBsAg positives. Hundred and six (40%) of them had multigravida, of whom 4 (1.5%) were HBsAg positives. Similar distributions of risk factors were observed from a study conducted in Western Ethiopia as 5.6% of them had dental procedures and 11.2% of them with tattoos [15].

## Conclusions

In conclusion, the prevalence of HBsAg among the delivering women in this study was 3.0%, which is an intermediate prevalence according to the WHO criteria that require routine screening and vaccination program. An almost equal rate of positivity in cord blood to the infected mothers (75%) revealed the need to have routine vaccination and maternal screening to prevent vertical transmission of HBV as previous history of routine ANC screening coverage was very low in these study sites (11%). The overall exposure status of mothers for possible risk factors was also high, which might also requires creating awareness using media in general and public health education in the health facilities in particular, as a priority prevention strategy.

However, due to a few number of HBsAg positive samples (both from the mothers and the cord bloods), conclusions from this study remains to be with caution. Therefore, large sample size studies and assessments for the neonatal transmission and possible risk factors including maternal knowledge, attitude and practice are also recommended.

## Abbreviations

ANC: Anti natal care; CDC: Centers for disease control and prevention; ELISA: Enzyme linked immuno sorbent assay; HBeAg: Hepatitis B e Antigen; HBsAg: Hepatitis B surface Antigen; HBV: Hepatitis B virus; HCC: Hepatocellular carcinoma; HCV: Hepatitis C virus; HIV: Human immunodeficiency virus; SOPs: Standard operating procedures; SPSS: Statistical package for the social sciences; WHO: World Health Organization.

## Competing interests

The authors declare that they have no competing interests.

## Authors’ contributions

All authors (DT, BT and KD) contributed to the design of the study and on the interpretation of the data. TT helped on laboratory testing technically. DT performed the data of analysis and drafted the manuscript. All authors (DT, BT, KD & TT) critically revised the draft manuscript and approved the final manuscript.
